# Baseline assessment of metal and microplastic pollution in sediments of a small-sized harbor in western Baltic Sea (Kalmar, Sweden)

**DOI:** 10.1007/s11356-025-36509-x

**Published:** 2025-05-22

**Authors:** Cheng Chang, Anna Augustsson, Changxun Yu, Rumbidzai Mugwira, Solomon Carlon, Marcelo Ketzer

**Affiliations:** 1 Linnaeus University Faculty of Health and Life Sciences: Linneuniversitet Fakulteten for halsö- och livsvetenskap, Kalmar, 391 82 Sweden; 2 Linnaeus University Faculty of Technology: Linneuniversitet Fakulteten for Teknik , Växjö, 351 95 Sweden; 3 Conservation International, Monrovia, 1500 Liberia

**Keywords:** Metal, Microplastic, Baltic Sea, Harbor, Sediment, Pollution

## Abstract

This study provides a baseline analysis of sediment pollution in Kalmar Guest Harbor, Sweden, focusing on metals and microplastics. The study site, a bustling coastal area, was chosen to investigate the connections between anthropogenic activities, bioturbation, and environmental contaminants. The results revealed that the pollution extends beyond 30 cm of depth below the seafloor, with elevated levels of copper (Cu), tungsten (W), cadmium (Cd), zinc (Zn), lead (Pb), and microplastics. Significantly, there is no previous publication alarming the W contamination in the Baltic Sea sediment, and therefore, our findings highlight the need for further investigation into tungsten contamination in the region. Furthermore, we explored the distribution patterns, potential sources and relationships of different contaminants. The K-means cluster analysis revealed that bioturbation is speculated to influences the depth concentration of pollutants, particularly at shallow depths (0‬–18 cm). Metal contaminants do not appear to be predominantly bound to MP particles.

**Graphical abstract:**

**Figure:**
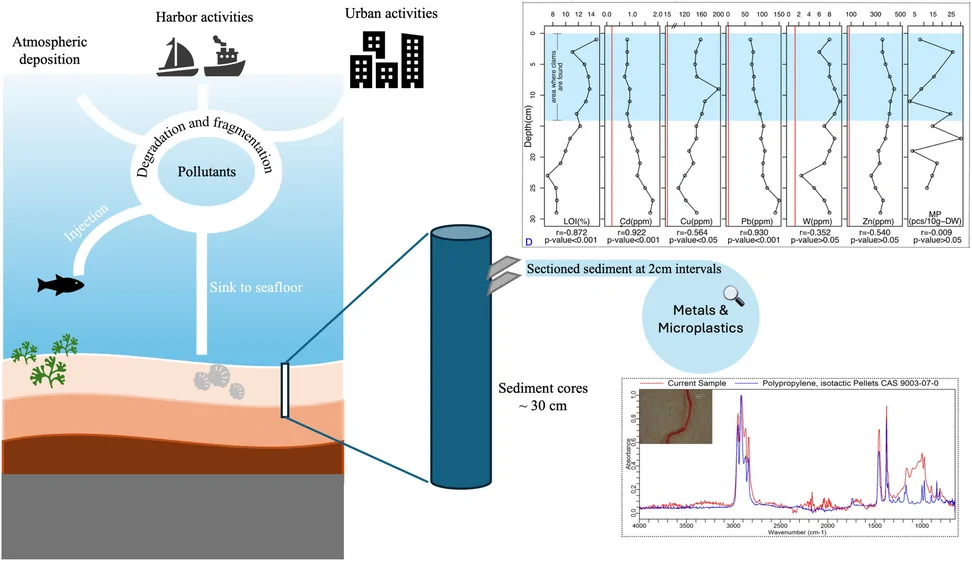

**Supplementary Information:**

The online version contains supplementary material available at https://doi.org/10.1007/s11356-025-36509-x.

## Introduction

Ocean and coastal sediments serve as habitats, nurseries, and feeding grounds for a diverse range of organisms, contributing to the overall health and functioning of the marine ecosystem (Snelgrove 1999). However, increasing contamination of these sediments by various pollutants poses significant short- and long-term threats to the marine ecosystem and human well-being (Johnston et al. 2015). As a result, the distribution, transport, environmental fate, and potential ecological effects of various pollutants in sediments have attracted considerable attention over the last decades (Yao et al. 2019; Shahabi-Ghahfarokhi et al. 2021b; Chiaia-Hernández et al. 2022).

Metals, for instance lead (Pb), cadmium (Cd), zinc (Zn), and copper (Cu), are among the most serious contaminants in aquatic ecosystems due to their high potential to enter and accumulate in the food chain (Maata and Singh 2008; El-Serehy et al. 2012). When these metals accumulate in the food web up to levels that are toxic to aquatic organisms, particularly predators, they may pose risks to both aquatic organisms and human health (Neff 2002; Tchounwou et al. 2012; Aytekin et al. 2019). Marine and coastal sediments act as both sinks and sources for metal pollutants, playing an important role in determining their transport, environmental fate, and ecological effects in the environment (Maata and Singh 2008). In coastal sediment, metal pollution can be attributed to various anthropogenic activities and natural processes, such as industrial and domestic sewage, agricultural activities, combustion of fossil fuels, and weathering of rocks (Park et al. 2020).

Beyond the metals listed above, tungsten was classified as an emerging environmental contaminant by United States Environmental Agency in 2008 due to its association with childhood leukaemia clusters (Hobson et al. 2020). The source of tungsten can be both natural or anthropogenic (Datta et al. 2017). Tungsten (W) has been widely used in industrial and manufacturing applications, e.g., in the production of cutting tools and drills, and light bulb filaments, due to its high density and tensile strength (Datta et al. 2017). The behavior of W in the environment is complex, and its solubility generally under alkaline conditions but can be influenced by intricate interactions with metals (e.g., iron and cobalt) and sediment/soil composition. Additionally, phosphorus has been shown to enhance W solubility by decreasing its sorption (Bolt and Mann 2016; Datta et al. 2017). Exposure to tungsten can cause various negative effects, including immunotoxicity, pulmonary toxicity and tumor promotion (Bolt and Mann 2016), and acute toxicity to fish (Strigul et al. 2010). While several studies have examined the distribution and behaviour of tungsten in Baltic Sea water (Bauer et al. 2018) and sediment (Kurzweil et al. 2022), to the best of our knowledge, no research has specifically addressed W contamination in Baltic Sea sediments relative to local reference values, underscoring a critical knowledge gap.

Plastics are another pollution concern in the marine environment. When smaller than 5 mm in size, they are commonly defined as microplastics (MPs) (Yao et al. 2019; Nikiema et al. 2020). Microplastics can be categorized into primary or secondary types, depending on their origins (Boucher and Friot 2017; Nikiema et al. 2020). Primary microplastics are directly produced as particles < 5 mm, such as abrasives in cosmetics, while secondary microplastics result from the fragmentation and degradation of larger plastic items (Boucher and Friot 2017; Nikiema et al. 2020). These tiny particles not only pose a physical threat to aquatic life but also act as carriers of harmful chemicals, potentially causing long-term negative effects on benthic community through the food chain (Gola et al. 2021).

Harbors, as dynamic interfaces between land and sea, are hubs of human activity and pivotal to the economic vitality of coastal regions. They facilitate international trade, fishing, and recreation, yet these activities also make them vulnerable to pollution. The influx of pollutants from urban runoff, industrial discharges, and maritime traffic can accumulate in harbor sediments, affecting water quality and marine life (Preston-Whyte et al. 2021; Dereszewska et al. 2023).

Globally, many studies have investigated the interactions and relationships between metals and MPs in oceanic and harbor sediments (Chen et al. 2007; Li et al. 2020; Patterson et al. 2020). However, such investigations remain scarce in the Baltic Sea. To address this gap, this study focuses on a typical harbor (the Kalmar Guest Harbor) along the western Baltic Sea coast, where sediments are strongly influenced by anthropogenic activities and benthic process. The aim of this study is to investigate the distribution patterns and potential sources of metal and MP pollutants in sediments of the harbor, with a particular focus on their relationships, interactions with organic matter, and possible effects of bioturbation. The results of this small-scale study can provide baseline data for further investigations in this region.

## Materials and methods

### Study site

**Fig. 1:**
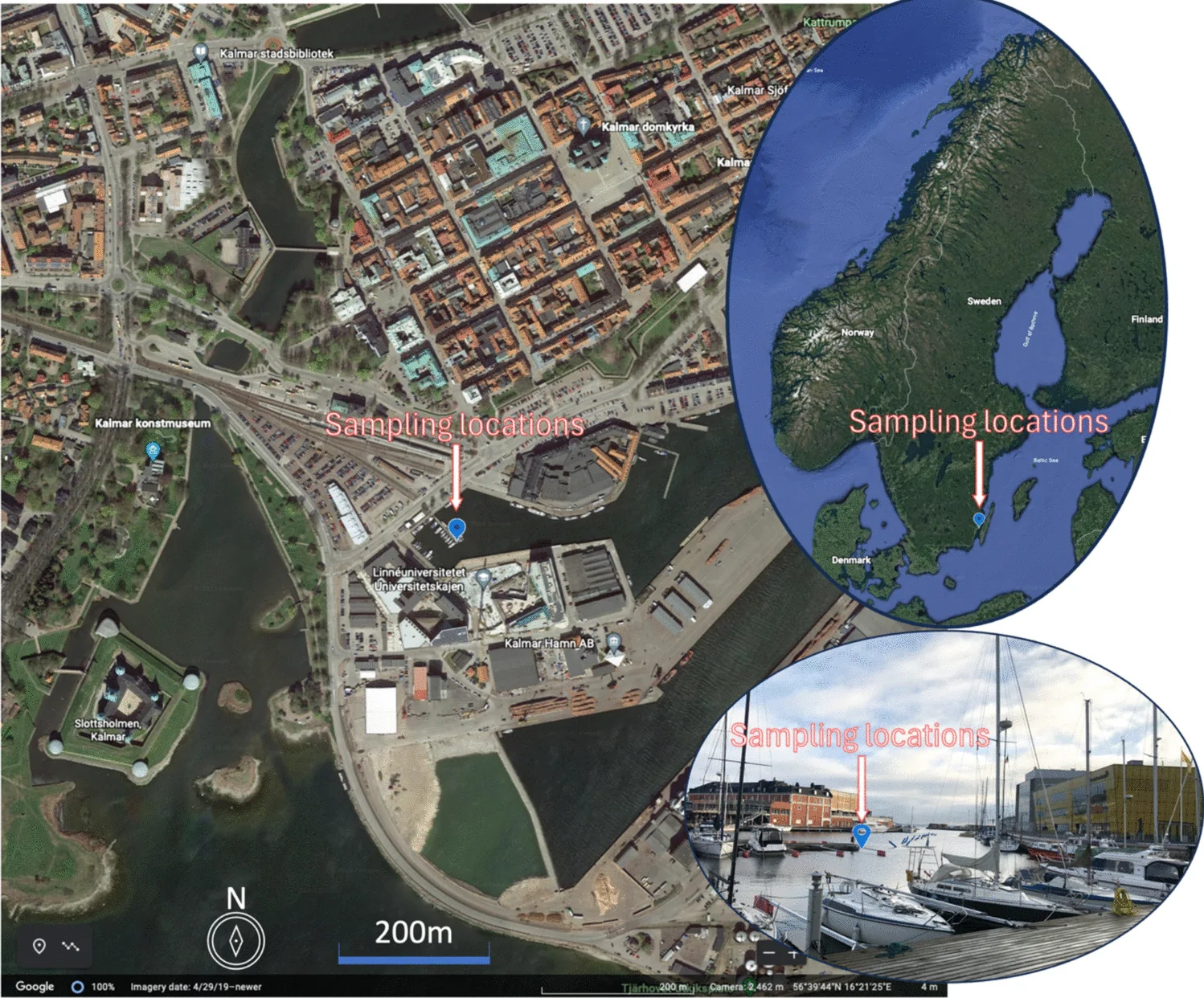
The location and a photo of the Kalmar Guest Harbor and sampling site. (Source: Google Earth)

The fieldwork was conducted at the Kalmar Guest Harbor, located on the western coast of the central Baltic Sea, as shown in Fig. 1. The Kalmar municipality has approximately 70,000 residents, with approximately 42,000 residents in the central urban area (Kalmar Kommun 2021). There are no domestic and industrial sewage discharges close to the sampling site. The harbor is open all year around and attracts mainly recreational boat, especially during summertime.

### Sediment sampling

On October 5, 2021, coring took place in the central part of the Kalmar Guest Harbor by a HTH Gravity Corer (Pylonex AB, https://www.pylonex.com). The water depth is around 3 m. Two representative sediment cores (30 and 26 cm long) were collected from the dock (Fig. 1, 56°39′44’’ N, 16°21′45″ E). The coring distance of two cores are around 1 m away from each other. The cores were sliced at two-centimeter intervals, resulting in a total of 15 samples for the analysis of organic matter and metal contamination (30 cm-long core) and 13 samples for the assessment of microplastic contamination (26 cm-long core). Following collection, the sediment samples were preserved in transparent clean polyethylene bags and stored in a refrigerator set to 4 °C until further use.

### Loss on ignition and metal concentration

The sediment samples were dried at 105℃. Loss on ignition (LOI, % dry weight) was determined by combustion of dried subsample in a furnace at 550 °C for 4 h (Heiri et al. 2001). The method has been proven to show strong correlations between LOI at 550 °C and organic carbon content, and is widely used in sediment studies (Heiri et al. 2001; Santisteban et al. 2004; Andersen and Krysell 2005; Stachowska et al. 2024).

Dried and pulverized subsamples were sent to an accredited commercial lab (Activation Laboratories Ltd. http://www.actlabs.com/), for multi elemental analysis. Briefly, 0.25 g of sediment was successively digested by four acids—hydrofluoric, nitric, perchloric, and hydrofluoric acids. The resulting suspension was subsequently heated to incipient dryness utilizing precision programmer-controlled heating in many ramping and holding cycles. Thereafter, *aqua regia* was used to bring samples back into solution. The concentrations of 23 elements (namely Al, As, Ba, Ca, Cd, Co, Cr, Cu, Fe, Ga, K, Li, Mg, Mo, Ni, Pb, Sc, Sr, V, W, Y, Zn, and Zr) in the solutions were determined by inductively Coupled Plasma Optical Emission Spectroscopy (ICP-OES). To ensure the accuracy of the analysis, quality control measures were implemented, including the use of reagent blanks, duplicate samples, and the analysis of 13 certified reference materials.

### Geoaccumulation index

The degree of metal contamination was assessed using the geoaccumulation index (I_geo_) (Muller 1969). The contamination was categorized into seven classes given the obtained I_geo_ values. The I_geo_ values were calculated based on the equation:

$${I}_{geo}={\mathrm{log}}_{2}(\frac{{C}_{i}}{1.5\times {C}_{b}})$$

where C_i_ represents the concentration of element *i* in the sample, and C_b_ denotes the background concentration level. The background concentration for each element adopted in this work referred to the criteria from the Swedish Environmental Protection Agency (Swedish EPA 2000). According to these criteria, the background values are set at the 50 th percentile of deep sediment samples (pre-industrial levels) from multiple areas along the Swedish coast. Additionally, the background values of metals in these sediments are relatively stable, although local variations may occur. The background concentrations of selected metals are provided in Table 2.

### Microplastic identification and characterization

The methods used to separate and characterize MPs in this study were modified from the NOAA guideline (Masura et al. 2015) and the standardized protocol (Frias et al. 2018) from JPI-Oceans (Joint Programming Initiative Healthy and Productive Seas and Oceans). The laboratory practical steps consisted of (i) sample drying at 50 °C until constant weight was achieved (i.e., completely dried sample); (ii) organic matter removal by slowly reacting the dry samples with 100 ml of 10% H_2_O_2_ for 24 h; (iii) sieving of the sediment and H_2_O_2_ solution through a stainless sieve with a mesh size of 20 µm and thoroughly rinsing the beaker and sieve with distilled water to transfer all residual solids to a beaker; (iv) density separation including addition of a saturated NaCl solution with a density of 1.2 g/cm^3^ and a volume of at least three times the volume of residual solids, and centrifugation of the liquid at 4300 rpm, 5–10 min for each sample (Tang et al. 2020; Grause et al. 2021; Xu et al. 2021); (v) filtration of the solution with paper filter with pore size < 15 μm and (vi) visual inspection of the filters with a stereomicroscope. The MPs were described taking into account their color, size, and shape. There is no universal definition to classify the shape of the microplastics (Kooi and Koelmans 2019), in this study we classify fibers as length to width ratio ≥ 3 (Kooi et al. 2021).

The polymer types of selected plastics (*n* = 29) were successfully analyzed by a Agilent Cary 630 FTIR (Fourier Transform Infrared) Spectrometer with ATR (Attenuated Total Reflectance) sampling module (Novillo et al. 2020). The plastics selected for analysis (n = 29) are those that could be easily picked up by hand and positioned on the ATR-FTIR, typically either long fibers exceeding 0.5 mm in length or fragments with large detection areas. The spectra were obtained under the specific conditions: 650–4000 cm^−1^; 32 scans; 8 cm^−1^ resolution. Samples’ spectra were compared with the reference spectra in the Agilent Polymers ATR library using the spectrometer's native software Agilent Microlab. Matches scoring are listed in the Supplementary Data-2.

#### Quality assurance and quality control

To avoid contamination, the laboratory procedures were conducted in a cleanroom with positive pressure. In addition, the samples were constantly protected using metal/glass covers or in a fume hood. To prevent airborne MP contamination during microscopic observations, the paper filters containing MPs were sealed with clean and transparent glass petri dishes.

Glass or metal tools were preferred, although transparent polyethylene bags, white rubber gloves and transparent polyethylene centrifuge tubes were utilized during operation. The density solution was filtered through a paper filter prior to use. Considering plastic materials utilized in the experiment (e.g., corer liner, plastic bag, gloves, etc.) as well as the potential airborne microplastic, three procedural blanks were conducted throughout the whole procedure, yielding an average of 2 plastic pieces per blank.

#### Limitation of microplastic analytical measurement

This study has several limitations that may influence the interpretation of the results. One key limitation is the use of density separation with a saturated NaCl solution, a cost-effective and widely used method in previous research (Van Cauwenberghe et al. 2015; Harris 2020). While effective, this approach is restricted to extract microplastics (MPs) with a density below 1.2 g/cm^3^, which may result in the exclusion of denser polymer types. Nevertheless, it efficiently captures the most commonly encountered polymers in the environment, such as polypropylene (PP), polystyrene (PS), and polyethylene (PE) (Frias et al. 2018; Phuong et al. 2021; Li et al. 2023).

Visual identification of microplastics (MPs) under a stereomicroscope can introduce both overestimation and underestimation errors. For example, natural fibers such as cotton and wool may be misclassified as MPs, leading to inflated counts (Xu et al. 2021). Conversely, smaller MPs that are challenging to detect visually might result in underreporting of their abundance. Even ATR-FTIR was employed in this study to confirm the chemical properties of some plastic pieces, most of the MP pieces are still not validated. In some studies (Uaciquete et al. 2024; Villanova-Solano et al. 2024; Watson and Turner 2024), particles identified through microscopy are termed “anthropogenic particles” unless chemically validated by advanced analytical techniques, including Laser Direct Infrared (LDIR), and Pyrolysis Gas Chromatography–Mass Spectrometry (Py-GCMS). They can offer greater accuracy in quantifying MP abundance and identifying their chemical composition (Dehaut et al. 2020; Cheng et al. 2022; Uaciquete et al. 2024).

Additionally, this study utilized white glass fiber paper employed during visual detection. This may have hindered the identification of anthropogenic particles of similar colors, potentially introducing bias against white and transparent particles.

Despite these limitations, similar analytical methods are still widely used in recent studies (Pervez and Wang 2022; Khan et al. 2023; Izlal et al. 2024; Souza et al. 2024) to assess MP abundance in the environment. This choice is often influenced by considerations such as cost-effectiveness and restricted access to advanced equipment.

### Statistical analysis

Pearson correlation analysis and K-means cluster analysis (KCA) were adopted to investigate the distribution of metal contaminants (Cd, Cu, Pb, W, and Zn) with depth. In addition, other elements (K, Al, Mg, Fe, and Ca) were included in our analysis due to their significant roles in overall environmental and sediment composition. Principal Component Analysis (PCA), hierarchical clustering analysis (HCA) and Pearson correlation analysis were applied to thoroughly investigate these relationships.

The data analysis was conducted using software R version 4.3.1. The normal distribution was assessed using the Shapiro–Wilk test (shown in Supplementary Data-1), and log transformation was applied to Cd concentrations to meet the normality assumption. To ensure comparability across different variables for KCA (using the ´*kms´* function with Euclidean distance method (Dai et al. 2018)), PCA (using the ´*prcomp´* function) (Xie and Wang 2020) and HCA (using the ´*hclust´* function) (Park et al. 2023), the data were standardized using the ´*scale´* function in R.

## Results and discussion

### Sediment characteristics and element composition

**Fig. 2:**
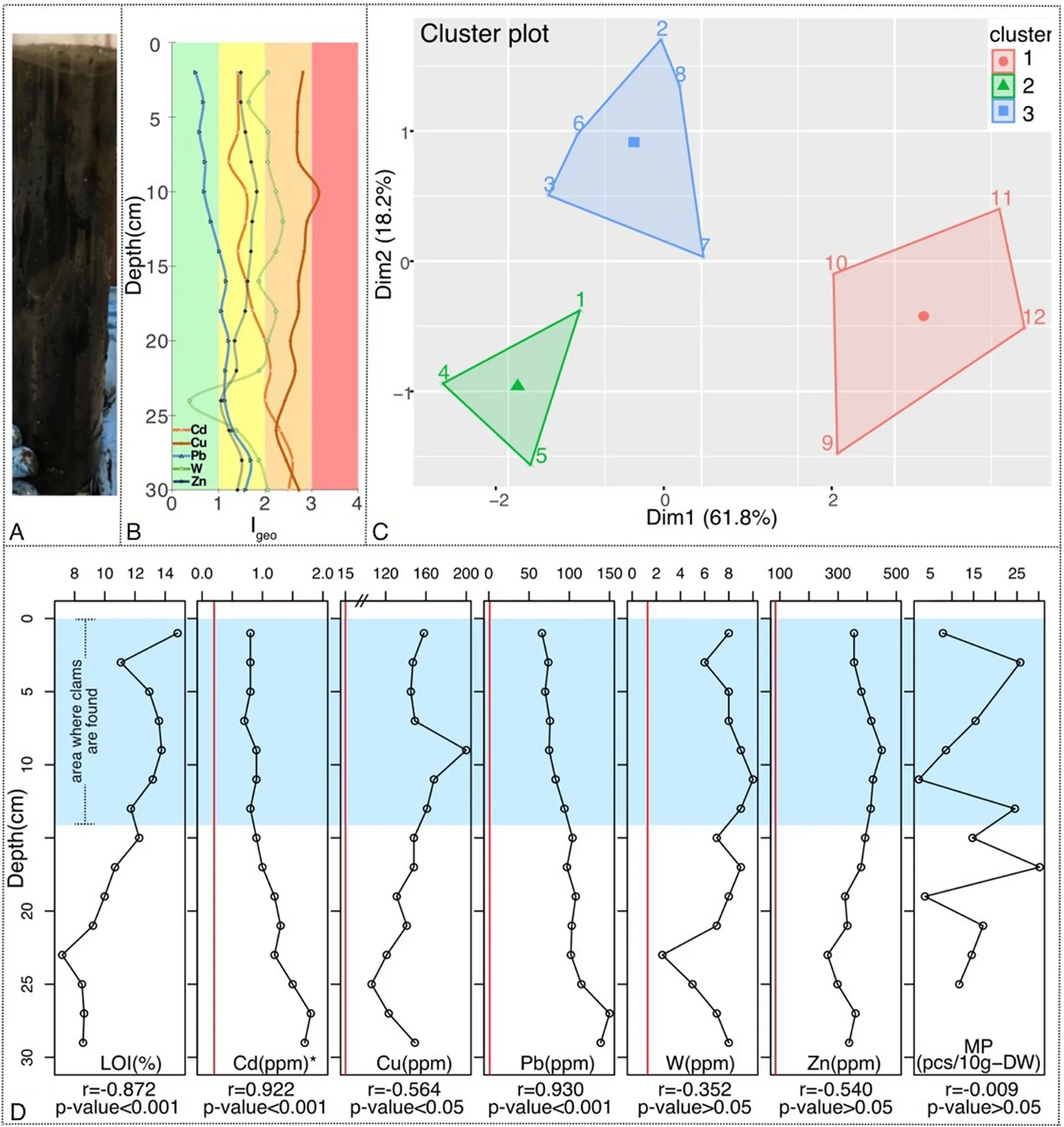
Contaminant distribution in sediment profile. (**A**) the picture of the collected sediment core. (**B**) Geoaccumulation Index (I_geo_) trends of metal contaminants with depth. (**C**) K-means Cluster Analysis of contaminants (Cd, Cu, Pb, W, Zn, and MP) based on depth. The numbers 1–12 in the plot shows the depth from surface to the deep: No. 1 (0–2 cm), No. 2 (2–4 cm), No. 3 (6–8 cm), No. 4 (8–10 cm), No. 5 (10–12 cm), No. 6 (12–14 cm), No. 7 (14–16 cm), No. 8 (16–18 cm), No. 9 (18–20 cm), No. 10 (20–22 cm), No. 11 (22–24 cm), No. 12 (24–26 cm). The data is grouped into three clusters. (**D**) Pearson Correlation analysis for the concentrations of contaminants and organic matter in relation to depth. The red line indicates the background level, and the blue shading marks the area with clams (0–14 cm)). *The concentration of Cd was subjected to natural log transformation to meet the assumption of normal distribution

The cores were predominantly composed of muddy sediment (Fig. 2A). Clams (*Macoma balthica,* around 1 ~ 2 cm) were frequently observed in the upper layers (< 14 cm) of the two sediment cores, suggesting strong activities of benthic communities and well-oxygenated conditions in the surface sediments (Adámek and Maršálek 2013). No other benthic invertebrates were found in the sediment cores.

The LOI values ranged from 7.2% to 14.8% (Fig. 2A), with a significant decrease with depth (*r* = −0.872, *p* < 0.001) due to degradability (Arndt et al. 2013). The surface layers exhibit a brownish-black coloration compared to the black hue observed in the deeper strata. The change in sediment color with depth may be indicative of geochemical transitions, such as the formation of iron monosulfides in deeper sediment layers where oxic conditions transition to reducing conditions (Myrow 1978; Giosan et al. 2002).

The concentrations of all detected elements of all samples are listed in Supplementary Data-1. Table 1 displays the values of average, median, minimum, maximum, standard deviation (SD), coefficient of variation (CV), skewness, kurtosis.

**Table 1:** Concentrations of detected elements in this study

	*Unit Symbol*	*Average*	*Median*	*Min*	*Max*	*SD*	*CV%*	*Skewness*	*Kurtosis*
*Al*	%	5,56	5,53	4,85	6,58	0,41	7,44	0,60	1,93
*As*	ppm	12,33	12,00	8,00	16,00	2,32	18,81	−0,15	−0,91
*Ba*	ppm	317,67	343,00	160,00	440,00	93,31	29,37	−0,40	−1,10
*Ca*	%	3,34	2,96	1,53	5,72	1,34	40,05	0,37	−1,22
*Cd*	ppm	1,09	0,90	0,70	1,80	0,35	32,23	0,97	−0,23
*Co*	ppm	9,07	9,00	7,00	11,00	1,03	11,39	−0,15	0,01
*Cr*	ppm	42,47	43,00	32,00	49,00	4,27	10,06	−0,81	1,37
*Cu*	ppm	146,33	148,00	106,00	200,00	22,04	15,06	0,55	1,82
*Fe*	%	3,42	3,54	2,85	3,78	0,31	9,04	−0,60	−0,97
*Ga*	ppm	13,07	13,00	11,00	14,00	1,03	7,90	−1,05	0,32
*K*	%	1,92	1,92	1,72	2,26	0,14	7,27	0,88	1,20
*Mg*	%	0,85	0,86	0,69	0,99	0,09	10,94	−0,34	−0,86
*Li*	ppm	24,13	25,00	20,00	27,00	2,53	10,49	−0,51	−1,20
*Mo*	ppm	2,03	2,00	0,50	4,00	0,93	45,97	0,95	1,66
*Ni*	ppm	25,40	26,00	21,00	29,00	2,35	9,27	−0,29	−0,70
*Pb*	ppm	97,07	97,00	66,00	150,00	24,61	25,35	0,80	0,21
*Sc*	ppm	9,00	9,00	7,00	10,00	0,93	10,29	−0,62	−0,18
*Sr*	ppm	249,47	237,00	208,00	327,00	38,40	15,39	0,77	−0,58
*V*	ppm	63,80	66,00	52,00	71,00	6,05	9,48	−0,77	−0,52
*W*	ppm	7,43	8,00	2,50	10,00	1,86	25,02	−1,39	2,60
*Y*	ppm	30,00	30,00	25,00	34,00	2,65	8,82	−0,45	−0,67
*Zn*	ppm	364,93	360,00	264,00	450,00	49,63	13,60	−0,28	−0,12
*Zr*	ppm	144,67	151,00	78,00	190,00	36,89	25,50	−0,48	−1,03

### Sediment contamination

#### Metal contamination

Through comparing the criteria from Swedish EPA with the concentrations of different elements in each depth (Supplementary Data-1), the results indicates that the sediment core exhibits moderate pollution (1 < I_geo_ < 2) to moderate-to-strong pollution (2 < I_geo_ < 3) by copper (Cu), tungsten (W), cadmium (Cd), zinc (Zn), and lead (Pb), as detailed in Table 2 with median values. These metals may pose long-term adverse effects on benthic communities within the harbor, and by extension, pose risks to human health.

**Table 2:** The I_geo_ values of the detected elements in the sediment core according to the Swedish national criteria (Swedish EPA 2000) and the classification criteria (Muller 1969; Shahabi-Ghahfarokhi et al. 2021a) based on the I_geo_ values

	Unit	Median	C_b_	I_geo_		Unit	Median	C_b_	I_geo_
As	ppm	12	10	−0,32	Ni	ppm	26	33	−0,93
Ba	ppm	343	371	−0,7	Pb	ppm	97	31	1,06
Cd	ppm	0,9	0,2	1,58	Sc	ppm	9	8,1	−0,43
Co	ppm	9	14	−1,22	Sr	ppm	237	168	−0,09
Cr	ppm	43	80	−1,48	V	ppm	66	104	−1,24
Cu	ppm	148	15	2,72	W	ppm	8	1,28	2,06
Ga	ppm	13	23	−1,41	Y	ppm	30	20	0
Li	ppm	25	52	−1,64	Zn	ppm	360	85	1,5
Mo	ppm	2	1,6	−0,26	Zr	ppm	151	190	−0,92
**I**_**geo**_	**Classification**			**label color**	**I**_**geo**_	**Classification**			**label color**
< 0	Unpolluted			white	3–4	Strongly polluted			red
0–1	Unpolluted to moderately			green	4–5	Strongly to extremely strongly polluted			black red
1–2	Moderately polluted			yellow	> 5	Extremely polluted			balck
2–3	Moderately to strongly polluted			orange					

The elements K, Al, Mg, Fe, and Ca are not included, considering the high level cannot be directly deemed as contamination. C_b_ refer to the background value of metal in Swedish coast sediments

Those contaminants (Cu, Cd, Zn, and Pb) have been found in many sediment studies in the Baltic Sea (Borg and Jonsson 1996; Fathollahzadeh 2013; Fathollahzadeh et al. 2014; Shahabi-Ghahfarokhi et al. 2021a, 2021b)- and other regions (Yunus et al. 2020; Al-Mutairi and Yap 2021). However, the contamination levels vary from site to site. A study (Shahabi-Ghahfarokhi et al. 2021a) across the Baltic Sea indicates that the highest concentration of Cu in sediment profiles at 13 different sites ranges from 45 to 200 ppm, while this study’s highest value is 200 ppm. Another study (Shahabi-Ghahfarokhi et al. 2021b) across the Baltic Sea indicates that the highest concentrations of Cd, Zn, and Pb in sediment profiles range from 0.4 to 15 ppm for Cd, 175 to 900 ppm for Zn, and 50 to 130 ppm for Pb, while this study’s highest values are 1.8 ppm for Cd, 450 ppm for Zn, and 150 ppm for Pb.

While the concentrations of Zn and Cu in the sediment fall within acceptable ranges for uses such as landscaping, due to their roles as essential trace elements to plant growth, it is imperative to regulate the levels of Pb and Cd (Senten 1989). Despite the sediment quality being suitable for less sensitive applications according to the Swedish national guidelines (Swedish EPA 2009), Pb and Cd are non-essential metals that warrant careful management. This is particularly true for Cd, which has a high potential for bioaccumulation, and thus requires stringent control to mitigate ecological risks (Senten 1989).

Moreover, it is noteworthy that the sediments are moderately to strongly contaminated with tungsten (I_geo_ = 2.06), identified by the US EPA in 2008 due to its severe biological impacts (Datta et al. 2017; Hobson et al. 2020). The presence of tungsten in the marine environment can lead to harmful effects, such as growth inhibition in aquatic plants (Strigul et al. 2005) and epithelial damage in various fish species (Datta et al. 2017). The absence of prior publications on tungsten contamination in Baltic Sea sediment underscores the novelty of this study. The detection of tungsten contamination herein, alongside other studies (Koutsospyros et al. 2006; Bolan et al. 2023), raises the alarm to examine the chemical status and potential ecological consequences of tungsten in the sediments of the Baltic Sea and its coastal areas.

#### Microplastic contamination

The sediment core contained a total of 427 MPs, along with 16 plastic pieces greater than 5 mm. The concentration of MPs in the entire sediment core was measured at 1553 pcs/kg-DW, with a range from 236 to 3023 pcs/kg-DW. The specific MP concentrations at various depths can be found in the Supplementary Data-1. Reviewing other studies carried out in the Baltic Sea helps to contextualize our findings within the broader state of knowledge on metal and plastic contamination in this region. The result is comparable to those reported for the other areas of the Baltic and North Seas. For instance, one study (Chubarenko et al. 2022) found that the upper 2*–*7 cm sediments from the Gotland, Gdansk, and Bornholm Basins (54 sites in total) on average contained 863 ± 1371 pcs/kg-DW MPs. Similarly, the surface sediments (no depth stated) from 21 stations in the Danish waters of the Baltic and North Seas were found to contain from 60 to 3600 pcs/kg-DW MPs (Strand et al. 2013). These findings align with the consensus that microplastics are pervasive in marine environments, often accumulating in significant quantities due to their persistent nature and widespread sources.

**Fig. 3:**
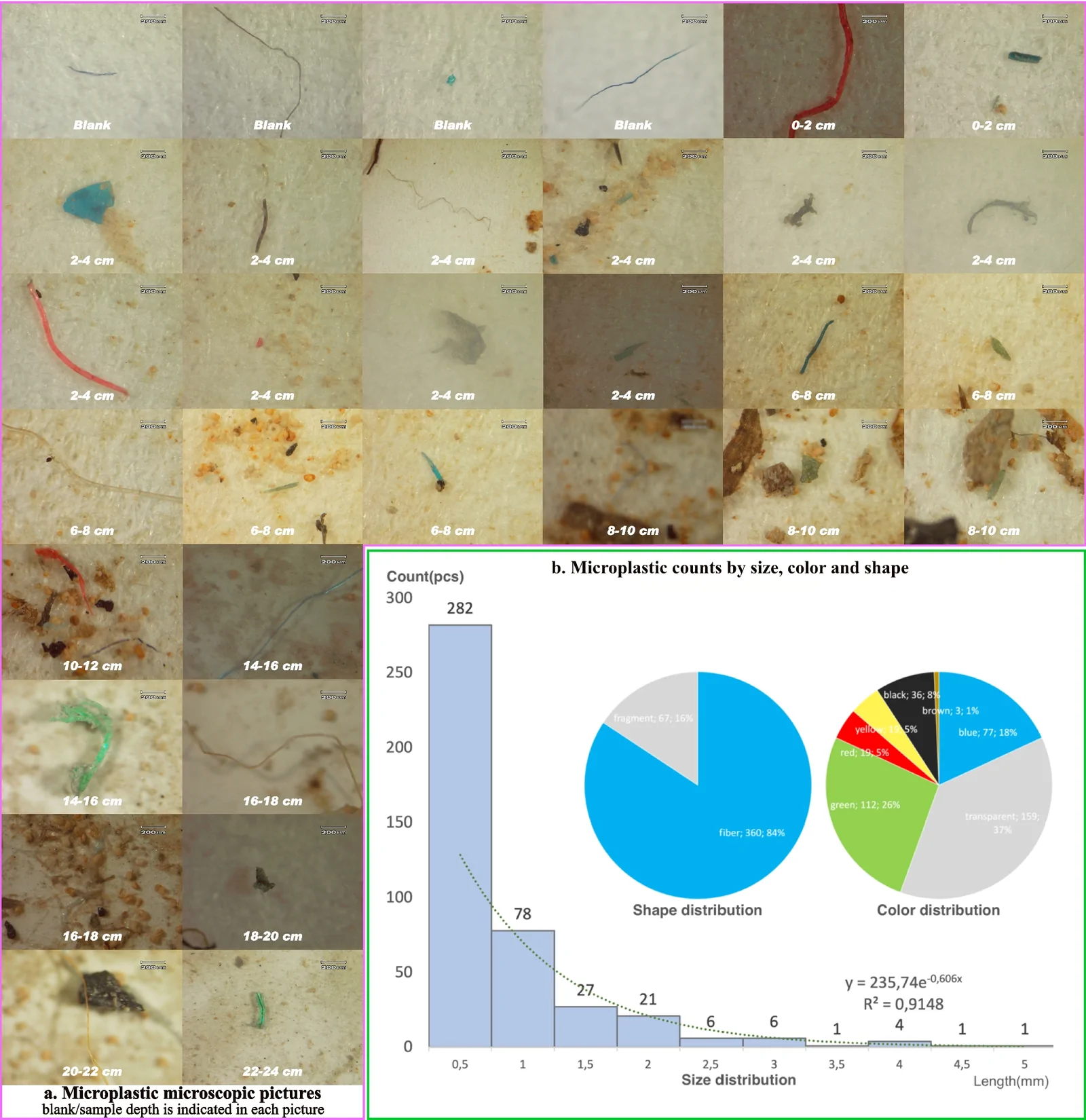
Typical microscopic images and characteristics of microplastics in the sediment core collected from Kalmar Guest Harbor. (**a**) Microscopic images of typical microplastics, with their corresponding depths indicated in white text within each image. (**b**) histogram illustrating the size distribution of microplastics, highlighting the greater abundance of smaller particles. The pie charts depict the composition of microplastics, categorized by shape (fiber or fragment) and color. Fibers are found to be the more prevalent in the samples than fragment. The most observed colors of the microplastics include transparent, green, blue and black

In terms of color, MPs predominantly appear to be transparent, with green, blue, and black also prevalent (Fig. 3). Transparent MPs are found to be the most abundant in some ocean and coastal sediments (Li et al. 2021; Chubarenko et al. 2022), while blue, black and green are also common colors for MPs in ocean and beach sediment (Stolte et al. 2015; Zheng et al. 2019; Yaranal et al. 2021; Zhou et al. 2021; Chubarenko et al. 2022). The color of MPs may play a significant role in their environmental accumulation and distribution patterns. This could be attributed to the differential rates of photoaging among colored MPs (Zhao et al. 2022), as well as the color preferences of aquatic organisms during ingestion (Martí et al. 2020).

The findings further reveal that smaller MPs (20*–*500 μm) were more abundant, accounting for about 66% of the total number of particles, and fibers were more prevalent, comprising 84% of the MPs. This is consistent with other studies (Li et al. 2021; Liu et al. 2023) indicating that smaller, fibrous MPs are common in sediments and carry heightened toxicity risks (Pirsaheb et al. 2020). Research indicates that the size and shape of microplastics (MPs) can affect their retention time, accumulation, and physical damage within organisms (Hussain et al. 2001; Jemec et al. 2016; Qiao et al. 2019). MPs up to 100 μm (dogs) or 150 μm (humans) have been found in lymph in dogs and humans, respectively (Hussain et al. 2001). More fibers have been found accumulated in the gut of fishes than fragment and bead (Qiao et al. 2019). The presence of such MPs in the harbor’s sediment is therefore concerning.

The analysis of the study particles revealed a total of 29 different types of plastics (Table 3). Among these, Nylon and polypropylene (PP) were the most prevalent polymers, each accounting for 6 pieces. Both plastic materials have been commonly found in coastal sediments, such as Belgian coast (Claessens et al. 2011) and the Adriatic Sea (Mistri et al. 2018). We also identified PE and PET in this study, which are common types of plastic found in ocean and coastal sediment (Yao et al. 2019; Nematollahi et al. 2020).

**Table 3:**
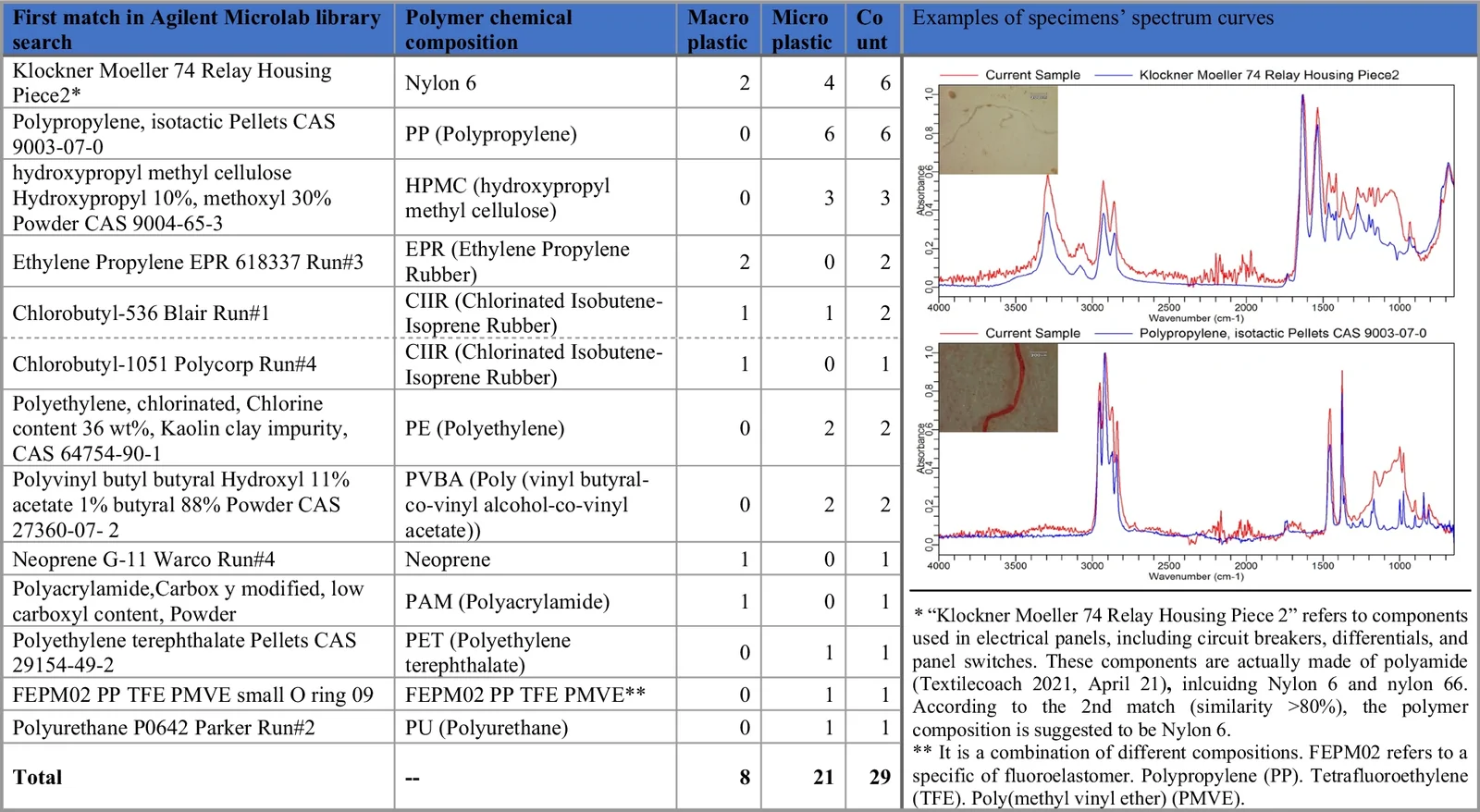
The polymer composition of plastic specimens (*n* = 29)

### Contaminant distribution patterns and controlling factors

#### Contaminant distribution patterns

Dredging is one of the most common and effective methods to improve environmental quality of harbors with contaminated sediments. The planning of dredging activities necessitates an examination of the distribution and thickness of contaminated sediments (Senten 1989). Our data showed that polluted sediments at the sampling site goes beyond 30 cm of depth (Fig. 2B).

Considering all determined contaminants comprehensively, the result of K-means Cluster Analysis is indicated in Fig. 2C, with the numbers 1*–*12 in the plot shows the depth from surface to the deep. The result shows that the samples are divided into three clusters. Cluster 1 only contains bottom layers (18*–*26 cm), while Cluster 2 and 3 correspond to upper layers (0*–*18 cm). Interestingly, the contamination characteristics of the surface layer (0*–*2 cm) are similar to the samples found at 8*–*12 cm depth, while the contamination characteristics of the 2*–*8 cm layer are more akin to the samples at 12*–*18 cm.

The distribution of contaminants in the sediment profile can be determined by multiple factors, including temporal variations of inputs, the speciation of pollutants, and sediment dynamics. Given the conditions of the sampling site described in 2.1, one explanation for the results is temporal variation of contamination inputs causing the stratification, while physical mixing processes in the sediment causes the mixture of cluster 2 and 3 in the upper layers (0*–*18 cm). Among the most common process contributing to the sediment mixing is bioturbation (Claessens et al. 2011; Outridge and Wang 2015). We therefore speculate that bioturbation may also be a relevant process influencing the contaminants distribution in the sediment profile in the study area. There are clams found in the upper layers (< 14 cm) which may contribute to the distribution of metal in the sediment profile through, for instance, burrowing, feeding and foraging. The area where clams were found is 4 cm shallower than the upper limit of cluster 3. It might be because the clams’ activities area is bigger than the area where the clams were found. In addition, the anchoring of ships and turbulence caused by deep draft vessels may also influence the metal distribution in sediment profile (Bothner et al. 1998).

However, the distribution of each pollutant in the sediment varies: The concentrations of W, Zn and microplastics (MP) do not show significant depth-related trends (W: *r* = −0.352, *p* > 0.05, Zn: *r* = −0.540, *p* > 0.05; MP: *r* = −0.009, *p* > 0.05). Conversely, the concentration of Cu shows a moderate negative correlation with depth (*r* = −0.564, *p* < 0.05). However, the Cd concentration exhibits the strongest positive correlation with depth (*r* = 0.922, *p* < 0.001), as well as Pb (*r* = 0.930, *p* < 0.001). This suggests that each pollutant distributes in a distinct manner. The decreasing concentrations of Cd and Pb with depth might reflect decreasing inputs of these two metals over time, even the sedimentation rate is not determined in this study. Meanwhile, these two metals have very similar depth profiles, meaning that they share the same sources and have similar geochemical behavior. Further insights into these relationships will be explored in the subsequent ‘Relationship Exploration’ section.

**Fig. 4:**
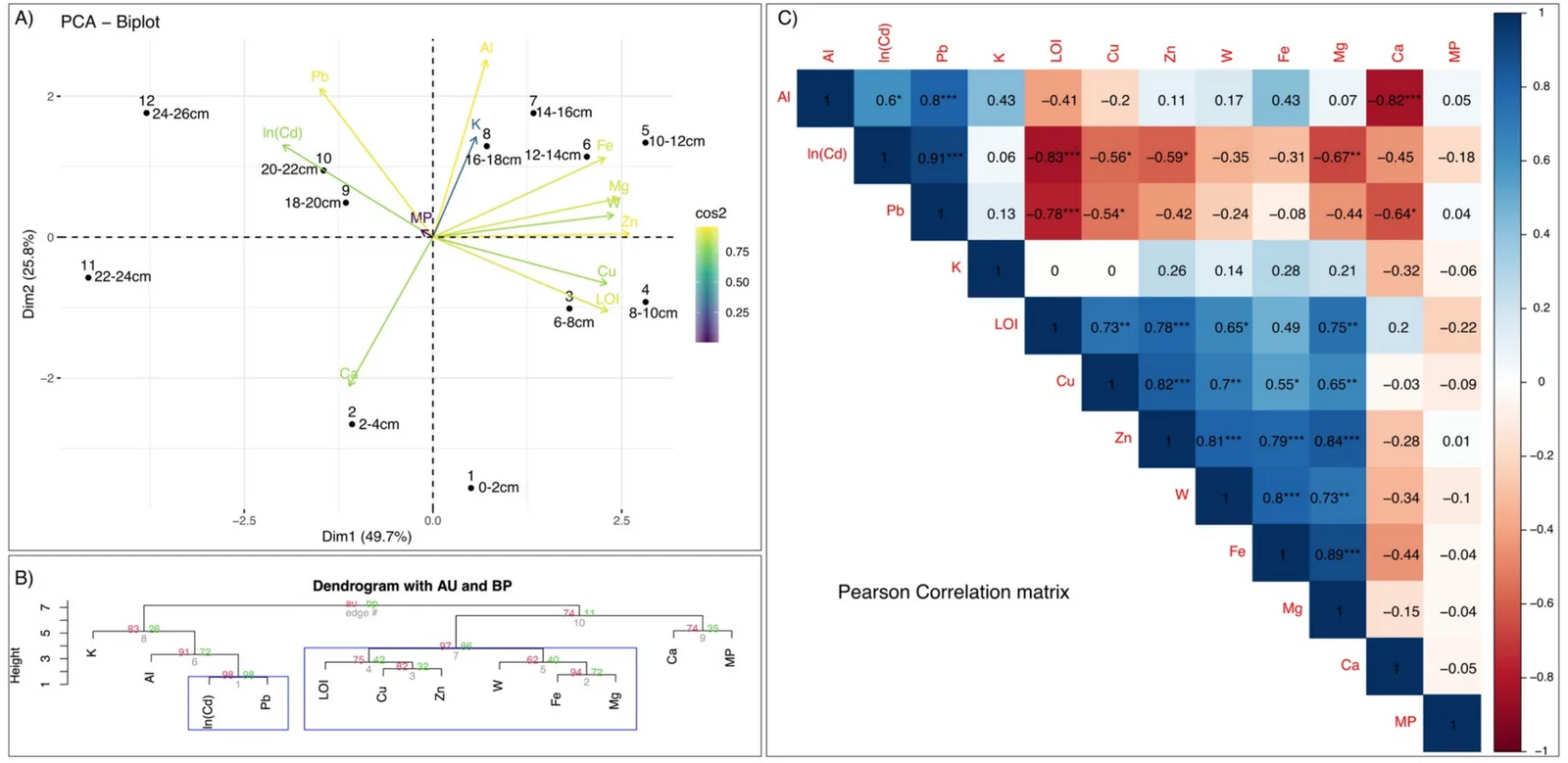
Principal component analysis (**A**), hierarchical clustering analysis (**B**), and Pearson correlation analysis (**C**) of the various elements, microplastics (MPs) and Loss on ignition (LOI) in the samples. The elements involved include primary contaminants Cd, Cu, Pb, W, Zn as well as K, Al, Mg, Fe, and Ca. (**A**) PCA biplot: This plot represents the first two principal components (Dim1 and Dim2) of the dataset, accounting for 49.7% and 25.8% of the total variance, respectively. The color scale labeled “cos2” indicates the quality of the representation of the elements on the biplot, with higher values indicating better representation. (**B**) Hierarchical clustering dendrogram: This dendrogram shows the clustering of observations. The height at which two objects are joined indicates the dissimilarity between clusters, with greater height representing larger dissimilarity. The analysis uses Euclidean distance and complete linkage method. AU (approximately unbiased) and BP (bootstrap probability) values are included to indicate the robustness of the clusters. Rectangles are used to highlight significant clusters. (**C**) Pearson correlation matrix: The heatmap displays pairwise correlation, with blue indicating positive correlation and red indicating negative correlation. Significance is indicated by asterisks (*** *p* < 0.001, **p < 0.01, *p < 0.05)

While the current dataset, which includes the concentration of elements and the types of plastics, doesn’t allow us to definitively pinpoint the origin of the contaminants, it does provide valuable insights. By analyzing this data and interpreting it in the context of our sampling points and relevant literature, we can begin to uncover potential sources and relationships of these contaminants. The results from the principal component analysis (PCA), hierarchical clustering analysis (HCA) and Pearson correlation analysis (Fig. 4) are utilized to explore the relationship of different contaminants.

#### Potential sources

The primary contaminants in the samples, namely Cu, Cd, Zn, and Pb as well as W (Table 2), may come from various sources. A literature review (Yunus et al. 2020) on the accumulation of heavy metals in coastal sediment have pointed out many potential sources. Specifically, the study harbor is a hub of activity, hosting a constant presence of yachts and boats, with a notable increase during the summer season. The sampling in this study was conducted on Oct.5, just after summer and there were still many boat activities. Given the absence of river and industrial effluents in the study harbor, the pollutants in the sediment could originate from atmospheric deposition, run-off from near-shore area, currents, accumulation of aquatic organism waste and remains, as well as activities related to boats (Chiaia-Hernández et al. 2022).

A study by the US EPA has found that Cu, Cd, Zn, and Pb are commonly present in various vessel discharges (US Environmental Protection Agency 2010). Activities such as boat maintenance, painting, and repair can generate a variety of these waste materials (Tech 2000), including debris from sanding, chips from antifoulant paint, scrap metal, pieces of fiberglass, sweepings, and lead and acid from batteries. Moreover, general boating activities and marina usage can lead to the generation of other solid waste items. These include bottles, plastic bags, coffee cups, cigarette filters, and fishing lines (Tech 2000).

Cu, which is most frequently found at toxic levels in marina waters, is commonly used as a biocide in antifoulant paints. Copper can leach into surface waters and sediments from bottom paints and scrapings (Tech 2000). Cadmium is used in batteries and brake linings (Tech 2000). Lead is used as a fuel additive and ballast and may be released through incomplete fuel combustion and boat bilge discharges (NCDEM 1991; Frederick 1995). Lead-acid batteries are another source of this pollutant (Tech 2000). Zinc anodes, which are used to prevent corrosion of metal hulls and engine parts, are another source of contamination. Zinc is also often found in motor oil and tires as well (Tech 2000). Tungsten applications span a wide range, including welding for metalworking, electrodes and contacts for electrical/electronic applications, super-alloys for turbine blades, as well as in the creation of fishing weights and jig heads (Koutsospyros et al. 2006).

The most common plastics identified in this study are made of Nylon and PP (Table 3). Nylon, a versatile material, is utilized in the production of various fibrous items such as apparel, carpeting, ropes, and airbags, as well as in the manufacture of fishing nets (Claessens et al. 2011). PP fibers are used in clothing, packaging, carpets and ropes which are used extensively on ships (Aizenshtein and Efremov 2006). Most of these plastic particles may originate from road runoff, discarded materials from boats, fishing nets, as well as atmospheric deposition.

#### Relationship exploration

The results of PCA, HCA, and Pearson correlation analysis reveal the relationships between different contaminants, as shown in Fig. 4. Elements Fe, Mg, W, Zn, Cu and LOI emerge as major contributors to the primary component in PCA results, which also locate together in the same branch of the HCA clustering dendrogram within height 3. In addition, the Pearson correlation result indicates positive linear correlations between contaminants W, Zn, Cu with LOI, Fe, and Mg, with statistical significance. Contaminants W, Zn and Cu may sorb onto sediment particles such as organic matter (humic material), and Fe-and Mn- oxyhydrides, precipitating or co-precipitating as relatively insoluble mineral phases (Outridge and Wang 2015). This observation aligns with findings from a study conducted in the Baltic Sea (Pempkowiak et al. 1999) and other coastal sediments (Okoro et al. 2012), which report that a significant proportion of Cu and Zn are bound to organic matter or Fe- and Mn- oxyhydroxides. Furthermore, samples collected from surface sediments (0–30 cm) in Fallon (USA) (Hobson et al. 2020) show that a large portion of W binds with organic matter.

Furthermore, the contaminants Pb and Cd exhibit a strong correlation (Fig. 4) and similar depth-dependent trends (Fig. 2), suggesting that these two contaminants may originate from common sources and undergo similar migration processes. The LOI, a measure of organic matter content, shows statistically significant negative correlations with the concentrations of Pb (*r* = −0.78, *p* < 0.001) and Cd (*r* = −0.83, *p* < 0.001) as depicted in Fig. 4C. This indicates that the presence of Pb and Cd is not associated with organic matter content. For instance, these contaminants may derive from sources, e.g., that are not rich in organic matter, they may not bind effectively with organic matter, or they may preferentially migrate into deeper sediment layers where organic matter content is lower.

In addition, microplastic contamination does not appear to have a significant correlation with the presence of metals and LOI in this study. Consistent results from previous studies (He et al. 2020; Nguyen et al. 2023) suggest either no correlation or a weak one between microplastic (MP) concentration and metals such as aluminum (Al), copper (Cu), iron (Fe), cadmium (Cd), zinc (Zn), and lead (Pb). These findings may imply that metal concentrations are more dependent on their sources rather than the concentration of microplastic (He et al. 2020). However, MPs are considered to work as vector of metals (Brennecke et al. 2016; Al-Mutairi and Yap 2021) and some studies (Maršić-Lučić et al. 2018; Li et al. 2020) have shown metal accumulation on microplastic particles. This implies a potential intricate interaction between the levels of metals and various other environmental aspects that affect their occurrence in sediment profile (Nguyen et al. 2023). A comprehensive study (Zhu et al. 2023) integrating 4984 experimental data points found that the adsorption capacities of MPs for heavy metals were influenced by several factors including the types of MPs and heavy metals, the adsorption environment, and the pH. In our specific sampling location, metal contaminants do not appear to be predominantly bound to MP particles. Further research is needed to fully understand the complex relationship between microplastics and metal contamination in sediments. This baseline study establishes foundational data for future monitoring and research efforts in this region.

## Conclusion

This study highlights the contamination by various metals (e.g., Cu, W, Cd, Zn, and Pb) and MPs in the sediments of Kalmar Guest Harbor. The contamination of tungsten in sediment, identified here for the first time in Baltic Sea, underscores the need for closer monitoring of this emerging contaminant. Bioturbation and anthropogenic activities, such as boating and fishing, are speculated to influence the distribution of pollutants. Although data were collected from a single sediment core without spatial replicates, these findings provide essential baseline data for understanding the interaction between metals and MPs in highly anthropogenically influenced harbor sediments. Future research should expand on these preliminary findings to explore contamination dynamics in other Baltic Sea regions and develop targeted strategies for mitigating pollution in coastal ecosystems.

## Supplementary Information

Below is the link to the electronic supplementary material. 

ESM1 (XLSX 21.0 KB) 

ESM2 (XLSX 20.0 MB)
